# A randomized translational study on protein- and glucose metabolism in skeletal muscles evaluated by gene-ontology, following preoperative oral carbohydrate loading compared to overnight peripheral parenteral nutrition (PPN) before major cancer surgery

**DOI:** 10.1186/s12967-024-05484-1

**Published:** 2024-07-22

**Authors:** Britt-Marie Iresjö, Ulrika Smedh, Cecilia Engström, Jan Persson, Christian Mårtensson, Kent Lundholm

**Affiliations:** 1 Institute of Clinical Sciences, Department of Surgery, Surgical Metabolic Research Lab, Sahlgrenska Academy at University of Gothenburg, Gothenburg, Sweden; 2 Department of Surgery, Region Västra Götaland, Sahlgrenska University Hospital, Gothenburg, Sweden

**Keywords:** ERAS, Preoperative nutrition, Skeletal muscle metabolism, Gene expression, Carbohydrate loading, Parenteral nutrition, mTOR signaling

## Abstract

**Background:**

Effects of preoperative drinks on muscle metabolism are unclear despite general recommendations. The aim of the present study was therefore to compare metabolic effects of a preoperative oral nutrition drink, recommended by protocols for enhanced recovery after surgery (ERAS), compared to overnight preoperative peripheral total parenteral nutrition (PPN) on skeletal muscle metabolism in patients aimed at major gastrointestinal cancer surgery.

**Methods:**

Patients were randomized, based on diagnosis and clinical characteristics, to receive either a commercial carbohydrate-rich nutrition drink (Drink); or overnight (12 h) peripheral parenteral nutrition (PPN) as study regimens; compared to isotone Ringer-acetate as Control regimen. Arterial blood- and abdominal muscle tissue specimens were collected at start of surgery. Blood chemistry included substrate- and hormone concentrations. Muscle mRNA transcript analyses were performed by microarray and evaluated for changes in gene activities by Gene Ontology algorithms.

**Results:**

Patient groups were comparable in all measured preoperative assessments. The Nutrition Drink had significant metabolic alterations on muscle glucose metabolism (p < 0.05), without any significant effects on amino acid- and protein metabolism. PPN showed similar significant effects on glucose metabolism as Drinks (p < 0.05), but indicated also major positive effects on amino acid- (p < 0.001) and protein anabolism (p < 0.05), particularly by inhibition of muscle protein degradation, related to both ubiquitination of proteins and autophagy/lysosome pathways (p < 0.05).

**Conclusion:**

Conventional overnight preoperative PPN seems effective to induce and support improved muscle protein metabolism in patients aimed at major cancer surgery while preoperative oral carbohydrate loading, according to ERAS-protocols, was ineffective to improve skeletal muscle catabolism and should therefore not be recommended before major cancer surgery.

*Trial registration* Clinical trials.gov: NCT05080816, Registered June 10th 2021- Retrospectively registered. https://clinicaltrials.gov/study/NCT05080816

## Introduction

Recent years, guidelines on preoperative fasting have been reconsidered to allow intake of clear fluids until few hours before surgery. Accordingly, the use of preoperative carbohydrate-rich drinks is recommended in Enhanced Recovery After Surgery (ERAS) protocols, implemented in clinical routines internationally, although evidence-based benefits are still unclear [1–3]. Systematic reviews and meta-analyses on preoperative carbohydrate loading have implied that carbohydrate supplementation is safe, and may or may not have benefits regarding morbidity, length of hospital stay or post-operative complications [4, 5]. Such discrepancies in outcomes may relate to differences in study designs with provisions of various oral drinks [4, 6], or that physiological and metabolic effects are actually less effective than expected [7].

Carbohydrate loading is used with purpose to counteract negative effects by short-term fasting, as depletion of glycogen stores and perhaps peripheral insulin resistance; with assumptions that such effects may reduce postoperative stress and improve whole body protein metabolism [7]. Reduction of post-operative insulin resistance by preoperative carbohydrate loading has been reported in some investigations [8–10], while others found no effects [11, 12]. However, few studies have evaluated effects on protein metabolism [13–15], which are most important for post-operative recovery and outcomes, particularly following major cancer surgery. It is highly unlikely that short-term carbohydrate provision should induce subsequent and overall positive effects on protein metabolism in heart, respiratory- and skeletal muscles in cancer patients, although some observations have suggested signs of improved post-operative muscle protein metabolism [12–14]. Therefore, the aim of the present study was to evaluate effects on skeletal muscle metabolism, in a three-group randomized study for proof of concepts. Patients scheduled for major gastrointestinal cancer surgery received either a preoperative nutrition drink (“standard carbohydrate loading”), as implemented in ERAS guidelines [1–3], or overnight infusion of peripheral total parenteral nutrition (PPN). Control patients received intravenous physiologic ringer-acetate only. Effects on skeletal muscle metabolism were evaluated by alterations in integrated gene expression patterns by microarray analyses (Gene ontology).

## Material and methods

### Patient inclusion and treatment interventions

**Fig. 1:**
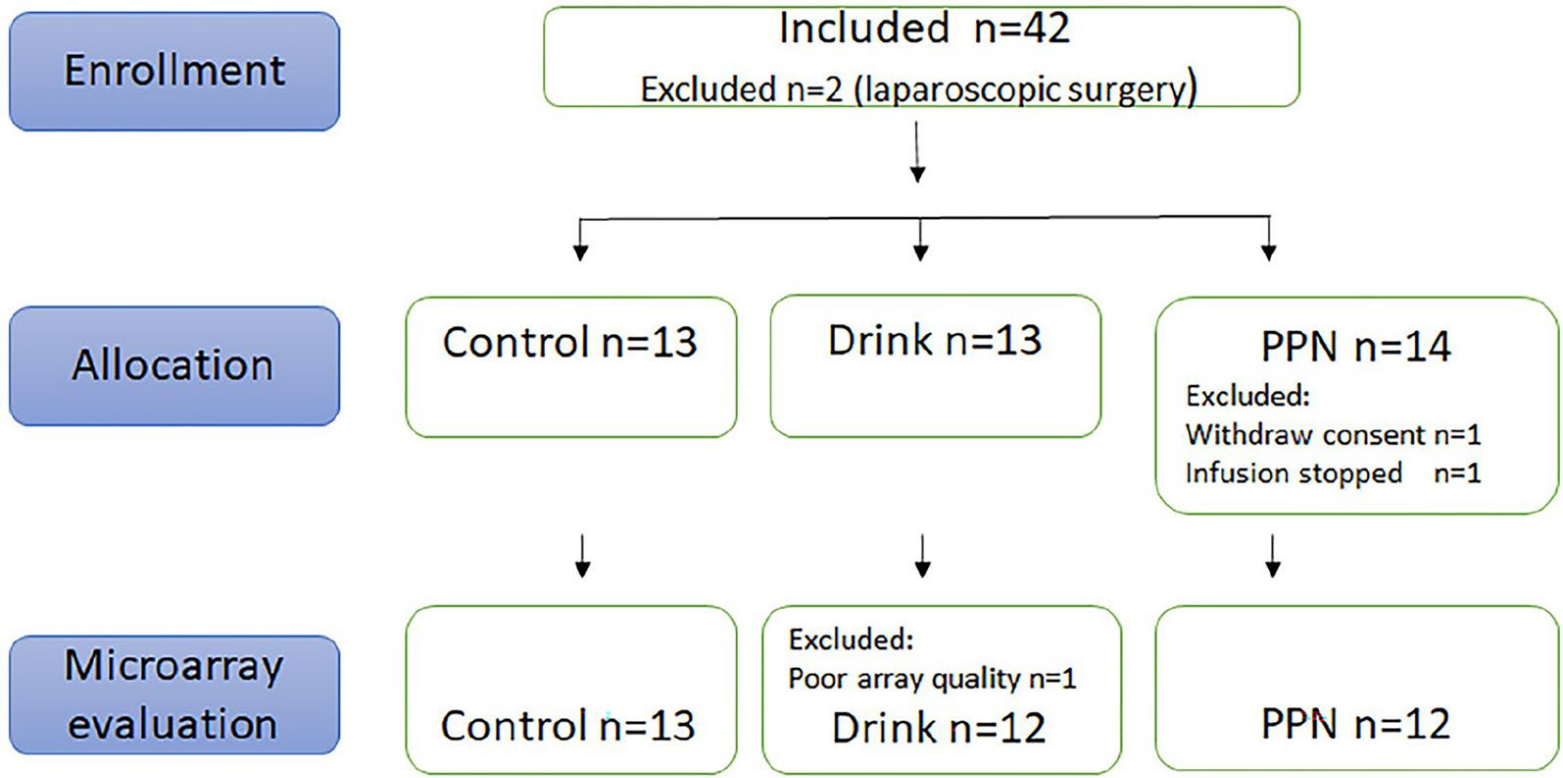
Flow chart of included patients after informed consent

Forty-two patients aimed at elective major surgery due to gastrointestinal cancer were asked to participate. Inclusion criteria were major open surgical procedures. Exclusion criteria were insulin dependent diabetes or steroid drugs. A study nurse or the physician in charge provided oral and written information and patient signed informed consent. Included patients were allocated to treatment groups by a digital algorithm [16], based on age, sex, body mass index (BMI), per cent weight loss relative to pre-disease weight, and type of cancer: esophageal, pancreatic or gastric cancer (See Flow chart, Fig. 1).

Study treatments were either a carbohydrate-rich nutrition drink (Drink) according to standard preoperative routine at the surgical clinic (Sahlgrenska Univ. Clinic) in agreement with ERAS routines [2, 3]; or overnight (12 h) peripheral total parenteral nutrition (PPN, cubital vein), as used in our previous studies [17, 18]; compared to intravenous (overnight 12 h) infusions of Ringer Acetate only (Control patients). Nutrition Drinks were provided as 400 ml of ProvideXtra^®^ Drink (Fresenius Kabi AB, Uppsala, Sweden; a product included in public procurement in Västra Götaland County, Sweden, serving a health care population of 1.7 million inhabitants), on the night before scheduled operation and 200 ml in the morning (06–07 am), 2–3 h before the start of surgery. PPN was provided at constant rate as all in-one bag solution, (SmofKabiven^®^Perifer, Fresenius Kabi AB, Uppsala, Sweden), at the infusion rate of 0.2 g N/kg/24 h equivalent to 1.67 ml/kg/hour). Ringer acetate was provided at constant rate by a pump (1.67 ml/ kg/hour). Infusions started preoperatively at 10 pm and continued for approximately 12 h until per-operative blood and muscle biopsies were obtained as initial surgical procedures. PPN provided ~ 400 kcal from carbohydrates, 180 kcal from amino acids and 350 kcals from fat (per 70 kg bodyweight, appendix 1). Drinks provided 804 kcal from carbohydrates, in the form of complex carbohydrates (maltodextrin and sucrose), and 96 kcal from protein (per total volume of 600 ml), while Ringer acetate contained zero calories (Appendix 1). Patient characteristics and results of preoperative venous blood tests were collected at the routine preoperative visit (Table 1). Fasting insulin was not included at pre-operative visits, since strict overnight fast was not allowed or requested immediately before major surgery according to the hypothesis in the present study. Therefore, we retrospectively matched our randomized patients to 109 cancer patients in our research database from earlier studies on over-night fasting cancer patients to confirm the probability that our present randomized study and control patients (Tables 1 and 2) had assumed similar insulin resistance as cancer patients in general, aimed for major cancer- or palliative surgery. The protocol was approved by the regional review board of Ethics in Gothenburg. Clinical trials.gov Registration number: NCT05080816.

**Table 1:** Patient and clinical characteristics at randomization and study inclusion

Patient characteristics and blood chemistry^a^	Ringer acetate (n = 13)	Drink (n = 13)	PPN (n = 12)
Male/Female, (n)	10/3	10/3	8/4
Type of cancer, (n) Esophageal/gastric/pancreatic	3/4/6	2/4/7	0/5/7
Age (Years)	69 ± 3	70 ± 3	69 ± 2
Weight at op (Kg)	75 ± 4	76 ± 5	71 ± 3
Height (cm)	176 ± 3	172 ± 3	174 ± 2
Body mass index	24.0 ± 0.7	25.2 ± 1.4	24.1 ± 0.8
Pre-disease weight	77.2 ± 4.2	84.1 ± 5.9	78.1 ± 4.0
Weight loss (%)	5.5 ± 1.5	8.3 ± 3.4	6.9 ± 2.3
Plasma glucose, mmol/L^a^	7.4 ± 2.0	6.9 ± 1.6	6.8 ± 1.1
Sodium, mmol/L	140 ± 1	138 ± 1	141 ± 1
Potassium, mmol/L	4.3 ± 0.1	4.2 ± 0.1	4.3 ± 0.1
Calcium, mmol/L	2.37 ± 0.03	2.32 ± 0.03	2.39 ± 0.04
Creatinine, µmol/L	79 ± 4	84 ± 6	86 ± 11
Protein, g/L	69 ± 1	68 ± 1	70 ± 2
C-reactive protein, mg/L	12 ± 5	12 ± 5	8 ± 3
Bilirubin, µmol/L	13 ± 5	39 ± 74	51 ± 24
ALP, µkat/L	2.5 ± 1	5.5 ± 3	5 ± 1
ASAT, µkat/L	0.6 ± 0.1	1.0 ± 0.4	2.7 ± 1.4
ALAT, µkat/L	0.6 ± 0.2	0.9 ± 0.3	2.5 ± 0.9

Mean ± SD, n = number of patients within parenthesis, without significant differences among the groups when comparing either body characteristics or blood and plasma variables

^a^Blood biochemistry was assessed on venous blood in a post-absorptive state. Fasting glucose and insulin was not achieved since strict over-night fast was not allowed or requested at standard pre-operative visits before the day of major surgery according to the hypothesis in the present study

The lack of fasting insulin levels for patients in Table 1 was compensated for by a data base matching procedure on 109 cancer patients, accounting for all patient variables in Table 1, which indicated average plasma insulin: 10.2 ± 0.7 mIE/L, and plasma glucose: 6.3 ± 2.4 mmol/L. This implies that our present patient material agrees with cohort patient information in our geographical region

**Table 2:** Arterial substrate concentrations when muscle biopsies were performed

Blood analyses	Ringer acetate (n = 13)	Drink (n = 13)	PPN (n = 12)	P <
Free fatty acids, mmol/L	0.68 ± 0.07	0.75 ± 0.07	0.54 ± 0.08	ns
Glucose, mmol/L	6.7 ± 0.3	5.6 ± 0.4	9.1 ± 0.4^a^	0.001
Glycerol, mmol/L	0.19 ± 0.06	0.14 ± 0.01	0.16 ± 0.02	ns
IGF-1, ng/ml	105 ± 11	105 ± 9	111 ± 10	ns
Insulin, mIE/L	11 ± 2	11 ± 3	22 ± 3^b^	0.05
Triglycerides, mmol/L	1.9 ± 0.2	1.7 ± 0.2	1.7 ± 0.2	ns
Urea, mmol/L	4.2 ± 0.4	5.8 ± 1.1	5.1 ± 0.4	ns

Patients were provided nutrition and infusions as described in Material and methods

Plasma was used in assays; glycerol in serum. *n*   number of patients within parenthesis. p-values indicate differences among patient groups by ANOVA

(Mean ± SE); ns not significant

^a^ p < 0.001;

^b^ p < 0.05 vs Ringer acetate (Control) and oral Drink

### Per-operative specimens

Muscle biopsies (rectus abdominis) were collected immediately at the start of surgery. Muscle biopsies were trimmed from any visible fat and placed in RNA later solution (within minute) to prevent RNA degradation. Biopsies were stored overnight at + 4 °C according to manufacturer’s instruction and thereafter kept frozen at −20 °C until RNA extraction. Arterial blood samples were collected shortly after muscle specimens were obtained. Blood samples were centrifuged at 1400 × g for 10 min, + 4 °C, within 30 min from collection. Plasma and serum samples were stored at −80 °C until analysis. Samples were coded and subsequently analyzed without knowledge of group allocation.

### Arterial blood analyses

Glucose, insulin, glycerol, triglycerides, free fatty acids, and urea were analyzed at the certified laboratory for Clinical chemistry at Sahlgrenska University hospital. Serum IGF-1 was determined by an IGF binding-protein blocked Radioimmunoassay (IGF-R22, Mediagnost GmbH, Reutlingen, Germany)*.*

Amino acids were quantified with the aTRAQ^™^ kit for Amino acid analysis of physiological fluids (AB Sciex) on a Sciex LC/MS/MS instrument in our research laboratory. Briefly, 40 µl plasma was deproteinized with 10 µl sulfosalicylic acid containing 4000 pmol of norleucine as internal standard (AB Sciex). 10 µl of the supernatant were labelled with the aTRAQ™ reagent according to manufacturer’s description (AB Sciex). Final amino acid concentrations were normalized relative to norleucine as the internal standard. Coefficients of variation of individual amino acid concentrations were 2.2–6.9%.

### RNA extraction and microarray hybridization

RNA was extracted with Qiagen fibrous tissue mini kit including optional DNAse step. RNA quantity and quality were checked in a Nanodrop one instrument (Thermo Scientific inc) and Agilent Bioanalyzer respectively. RIN values were within the range of 6.9–8.0, except for two samples with RIN values at 6.1 and 6.4.

### Microarray hybridization

Total RNA from each sample (200 ng) was labelled and amplified with Cy3-dCTP using Agilent Low Input Quick Amp Labelling kit according to the manufacturer’s protocol. Samples were spiked with Agilent One-Color Spike-Mix (1:10). The labelled and amplified cRNA was purified using the Qiagen RNeasy mini spin kit. Purified cRNA was quantified using a NanoDrop spectrophotometer. All samples had cRNA yields > 0.825 µg and Cy3 specific activity > 6.0. Samples were hybridized to Agilent SurePrint G3 Human GE v3 8 × 60 K Microarrays and incubated under rotation at 65 °C for 17 h. After hybridization, arrays were washed according to manufacturer’s protocol and immediately scanned using Agilent DNA microarray scanner G2505C at 3 µm resolution (Agilent Technologies). The fluorescent intensities of scanned images were extracted and preprocessed with the Agilent Feature Extraction Software (version 10.7.3.1). Sureprint G3 Human gene Expression v.3, 8 × 60 K arrays covers all human coding genes, plus non-coding transcripts, including lnc RNA and miRNA transcripts (total 58341 entities).

### Data processing by genespring software v.14.9.1

Feature extraction pre-processed files were imported to Genespring software with standard flag settings. (Population outlayers/saturated features/non uniform features = compromised; not above background and not positive and significant = not detected). One array file was excluded from analyses at quality control due to non-even distribution of green signal. Subsequently, final microarray evaluation included 37 muscle samples (13 Controls, 12 Drinks, 12 PPN), (Flowchart, Fig. 1). Files were filtered to remove compromised and not detected signals, from each array. Thus, the filtered list contained only non-compromised transcripts with detected signal, (i.e. a signal significantly above background signal, > 2.6 SD) in all arrays (21232 entities of 58341 remained after filtering). ANOVA tests (p < 0.05), followed by Newman-Keuls post-hoc testing, were used to generate the final dataset for analyses to map biological functions as described in the results. In total, 1217 entities passed ANOVA test at p < 0.05 and were followed by Newman Keuls Post Hoc test to indicate significant differences among patient groups; (Drink vs. Control: 508 transcripts; PPN vs. Control: 619 transcripts; PPN vs. Drink: 590 transcripts).

### Statistics

Pre-study power calculation indicated a need of 10 patients per group to detect 1.25-fold alterations in gene expression (α-5%, power 80%). One patient withdraw consent to participate for private reasons, two patients were excluded from the study due to laparoscopic instead of open surgical procedure, and one patient since the PPN infusion was accidentally stopped before operation start. Muscle biopsies were obtained from 12 patients in the PPN group, 13 patients on Drinks and 13 patients as Controls (Fig. 1).

Results are presented as mean ± SEM. One-way ANOVA followed by LSD post hoc test (parametric) were used to detect differences among the patient groups in amino acid and blood analyses. p < 0.05 was considered statistically significant in two tailed tests. ANOVA (Analysis of variance) evaluates effects of independent variables that may be either nominal or continuous. The statistical significance is determined by the comparison of variance among groups or repeated measures. Thus, variable requirements under test must not represent normal distribution, but should have equal distributions, which is a benefit in multi-group analysis on limited number of observations. Tibco Statistica v 13.4.0.14 was used for statistical tests.

Statistical evaluations on microarrays were performed in Genespring v 14.9.1 (Agilent). Significance level at p < 0.05 was regarded statistically significant by ANOVA, accounting for multitesting provided by the computer program, followed by Newman Keuls post-Hoc tests (non-parametric) for subsequent comparisons among groups. Gene Ontology and Pathway analyses were considered at p-values < 0.1 with correction for multiple testing.

## Results

### Peri-operative blood concentrations

Patient demographics and preoperative venous blood samples are reported in Table 1, with emphasis that strict overnight fasting state was not requested. Per-operative, arterial plasma glucose and insulin concentrations were significantly increased by PPN as expected compared to Drinks and Ringer acetate infusions (Controls), (p < 0.05, Table 2). Serum glycerol, plasma triglycerides, free fatty acids, IGF-1 and urea were comparable among patient groups (Table 2).

The sum of arterial plasma (proteinogenic) amino acids increased by PPN infusion as expected, but was unchanged by Drinks; (+ 81% vs. −3.4%) compared to Controls. PPN infusions increased branched chain amino acids (BCAA), while Drinks unfortunately decreased BCAA compared to Controls [19–21]. All other amino acids remained unchanged by Drinks compared to Control infusions (Table 3). Arterial concentrations of Tyrosine did not change by any treatment (Table 3).

**Table 3:** Arterial plasma amino acid concentrations when muscle biopsies were performed as the first procedure following start of surgery

	Ringer acetate (n = 13)	Drink (n = 13)	PPN (n = 12)	P-value <
Sum of all AA	2443 ± 101	2359 ± 116	4442 ± 793^a^	0.001
Sum of essential AA	687 ± 32	627 ± 39	1256 ± 55^a^	0.001
Sum of BCAA	324 ± 22	258 ± 19 ^c^	519 ± 24^a^	0.001
Essential AA				
Isoleucine	67 ± 6	56 ± 4	123 ± 7^a^	0.001
Leucine	56 ± 5	43 ± 4	81 ± 5^a^	0.001
Valine	201 ± 12	159 ± 12^c^	314 ± 14^a^	0.001
Lysine	178 ± 8	178 ± 10	272 ± 14^a^	0.001
Methionine	16 ± 1	16 ± 1	68 ± 4^a^	0.001
Phenylalanine	47 ± 2	48 ± 3	99 ± 4^a^	0.001
Threonine	98 ± 8	103 ± 10	250 ± 16^a^	0.001
Tryptophane	24 ± 2	23 ± 2	47 ± 3^a^	0.001
Non-essential AA				
Alanine	253 ± 25	255 ± 25	511 ± 48^a^	0.001
Arginine	53 ± 4	58 ± 4	146 ± 9^a^	0.001
Aspartic acid	4 ± 1	4 ± 0.3	4 ± 1	ns
Asparagine	51 ± 2	51 ± 2	45 ± 4	ns
α-abu	18 ± 2	13 ± 1	31 ± 3^a^	0.001
Citrulline	31 ± 2	33 ± 2	41 ± 3^b^	0.05
Cystein	60 ± 6	59 ± 4	59 ± 7	ns
Glutamic acid	73 ± 8	95 ± 28	99 ± 12	ns
Glutamine	694 ± 29	622 ± 54	873 ± 39^b^	0.001
Glycine	194 ± 15	219 ± 20	518 ± 49^a^	0.001
Histidine	72 ± 2	66 ± 4	112 ± 5^a^	0.001
Serine	102 ± 5	99 ± 5	255 ± 18^a^	0.001
Taurine	70 ± 7	86 ± 14	110 ± 7^c^	0.05
Tyrosine	46 ± 3	53 ± 4	47 ± 2	ns
Ornitine	71 ± 7	79 ± 8	146 ± 9^a^	0.001
Proline	153 ± 12	151 ± 13	517 ± 74^a^	0.001

Patients were provided nutrition and infusions as described in Material and methods

(Mean ± SE). *n*   number of patients, *ns*   not significant, *AA*   amino acids

p-values in table indicate differences among patient groups by ANOVA. Superscript letters indicate between group differences by ANOVA post-hoc-tests

^a^ p < 0.001 vs Drink and Control

^b^ p < 0.01 vs Drink and Control

^c^ p < 0.05 vs Control

### Overall alterations in muscle tissue gene expression among patient groups

**Fig. 2:**
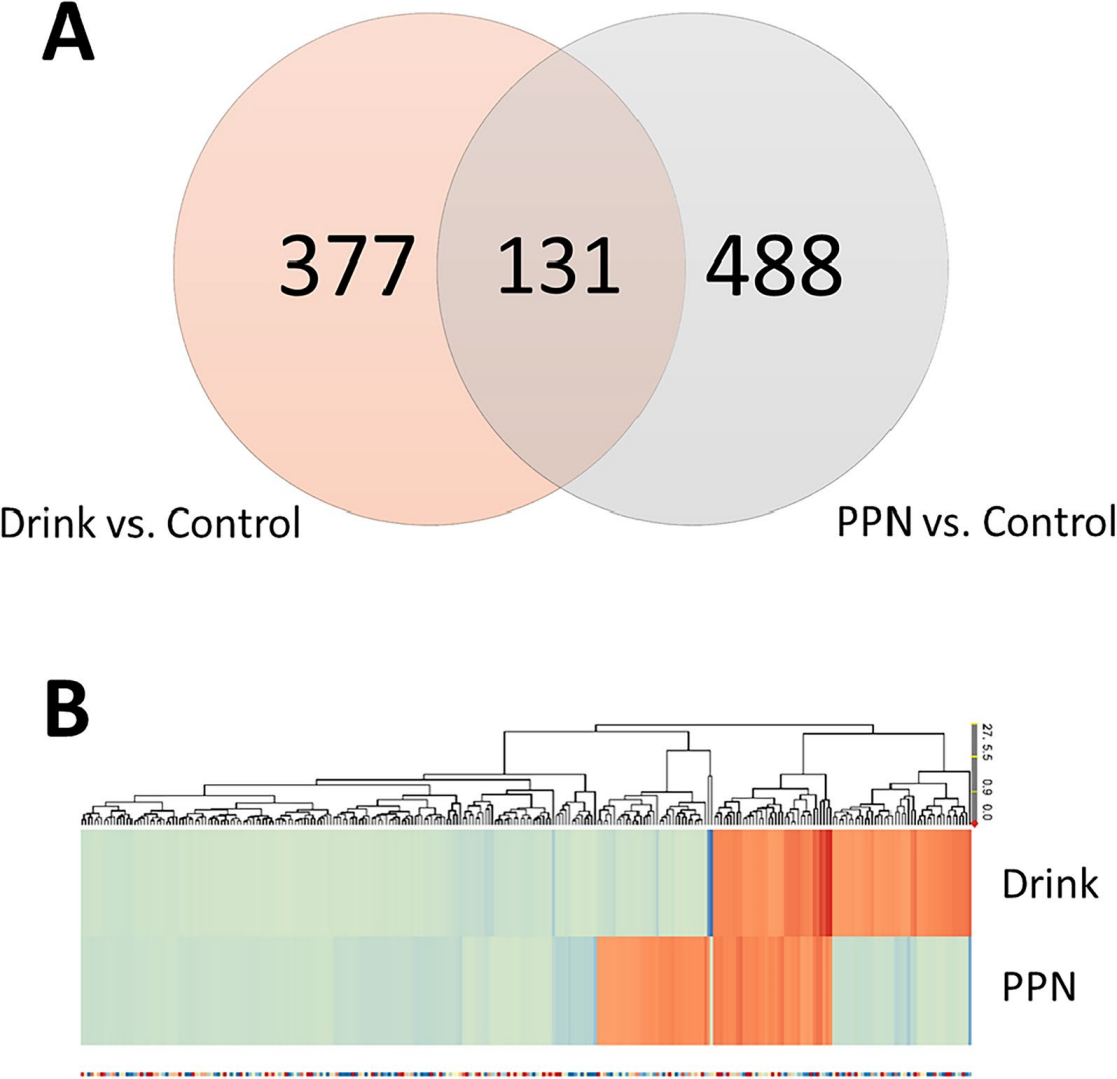
**A** Number of significantly altered transcripts in skeletal muscle biopsies from nutrition treated patients compared to control patients (Ringer acetate, p < 0.05). The Venn diagram illustrates that majority of alterations are specific to each nutrition treatment. Study patients received either a preoperative nutrition drink (carbohydrate loading), or overnight total parenteral nutrition as described in Material and methods. **B** Cluster analysis on significantly altered transcripts (ANOVA p < 0.01). Values are Fold Changes in study patients (Drink, PPN) compared to Control patients (Ringer acetate). The majority of altered transcripts showed similar directional changes of regulation in both study groups (Drink, PPN), while two sets of transcripts displayed inverse regulation (orange = up-regulated, blue = down-regulated) compared to Control patients (Ringer acetate)

ANOVA analyses among the patient groups indicated 1217 transcripts as altered (p < 0.05). Post-hoc analyses (Newman Keuls) showed that both Drinks and PPN induced approximately 500 statistically significant transcript alterations compared to Controls (Fig. 2A). Cluster analysis showed that the majority of transcript alterations displayed similar directional changes by Drinks and PPN, either up- or down-regulated, compared to Control patients (Fig. 2B). However, a sub-set of transcripts showed inverse pattern of changes with up-regulation by Drinks and down regulation by PPN; or vice versa when compared to Control patients (Fig. 2B). Transcripts with the largest fold change by treatment alternatives are reported in Table 4. These transcripts relate to diverse functions. Some up-regulated genes by Drinks were associated with transcriptional regulation and cell division (RFX2, CENPM), while down-regulations were obvious in transcripts of genes, located in the hemoglobin gene cluster (HBG1, HBQ1, HBA2, HBD). TBC1D1-transcripts, a gene involved in glucose transport and GLUT4 translocation, were among the most down-regulated by Drinks. CISH, with related suppression of cytokine signaling genes, were > fourfold up-regulated by PPN. Also, transcripts of MYH1 gene, coding for the fast isotype Myosin heavy chain protein 2X, were down-regulated, consistent with our previous findings [18]. Some transcripts with largest alterations by PPN infusions have yet unknown functions (Table 4).

**Table 4:** RNA transcript alterations among patient groups with fold change > 2

Gene name	Known or proposed function*	Gene symbol	Drink vs. control	PPN vs. control	PPN vs. drink	Gene bank accession number
*Oral drink*						
Regulatory factor X2	Transcription activiator	RFX2	**2.4**	1.1	**−2.2**	NM_000635
Tubulin polymerization promoting protein family member 3	Enables tubulin binding activity	TPPP3	**2.4**	1.3	**−1.8**	NM_016140
Centromere protein M	Kinetochore protein; involved in cell division	CENPM	**2.1**	1.5	−1.4	NM_024053
Hemoglobin subunit gamma 1	Oxygen transport, enriched in macrophages	HBG1	**−5.4**	1.0	**5.0**	NM_000559
Hemoglobin subunit theta 1	Oxygen transport, immune response	HBQ1	**−2.8**	−1.1	**2.5**	NM_005331
Hemoglobin subunit alpha 2	Oxygen transport	HBA2	**−2.4**	−1.3	**1.9**	NM_000517
Hemoglobin subunit delta	Oxygen transport	HBD	**−2.4**	−1.3	1.9	NM_000519
Vanin 2	Vitamin B5 metabolism	VNN2	**−2.2**	−1.2	1.8	NM_004665
Formyl peptide receptor 1	Chemotactic response	FPR1	**−2.1**	−1.2	1.8	NM_002029
Nuclear factor, erythroid 2	Inflammatory response	NFE2	**−2.0**	−1.3	1.5	NM_006163
TBC1 domain family member 1	GLUT 4 translocation	TBC1D1	**−2.0**	−1.5	1.3	XR_925212
*PPN infusion*						
Cytokine inducible SH2 containing protein	Suppression of cytokine signaling genes	CISH	2.0	**4.5**	2.3	NM_145071
KCNQ5 intronic transcript 1	Non-coding; possible lncRNA	KCNQ5-IT1	1.2	**2.1**	1.7	NR_120503
Leucine rich repeat containing 3B	Tumor suppressor/cell proliferation	LRRC3B	1.7	**−3.4**	**−5.7**	NM_052953
Chromosome 1 open reading frame 158	Known as CFAP 107. Unknown function	C1orf158	1.2	**−2.5**	**−3.0**	NM_152290
Solute carrier family 25 member 34	Mitochondrial protein transporter	SLC25A34	−1.0	**−2.4**	**−2.4**	NM_207348
Myosin heavy chain 1	Skeletal muscle protein; fast isoform	MYH1	−1.6	**−2.3**	−1.5	NM_005963
Tetraspanin 8	Cell surface protein. Complexes with integrins	TSPAN8	1.0	**−2.1**	**−2.1**	NM_004616
Novel Transcript	Unknown		−1.6	**−2.1**	−1.2	ENST00000417782

*Functions were assigned according to functions listed by either NCBI gene, the Human Proteome Atlas or literature search

Average FC values are reported when multiple probes were present on microarray

ANOVA among groups, p < 0.05 and FC > 2 followed by Student-Newman Keuls post hoc p < 0.05

Statistically significant difference among indicated treatment groups (p < 0.05) are indicated by bold font. Positive and negative values indicate up-or down-regulation

**Fig. 3:**
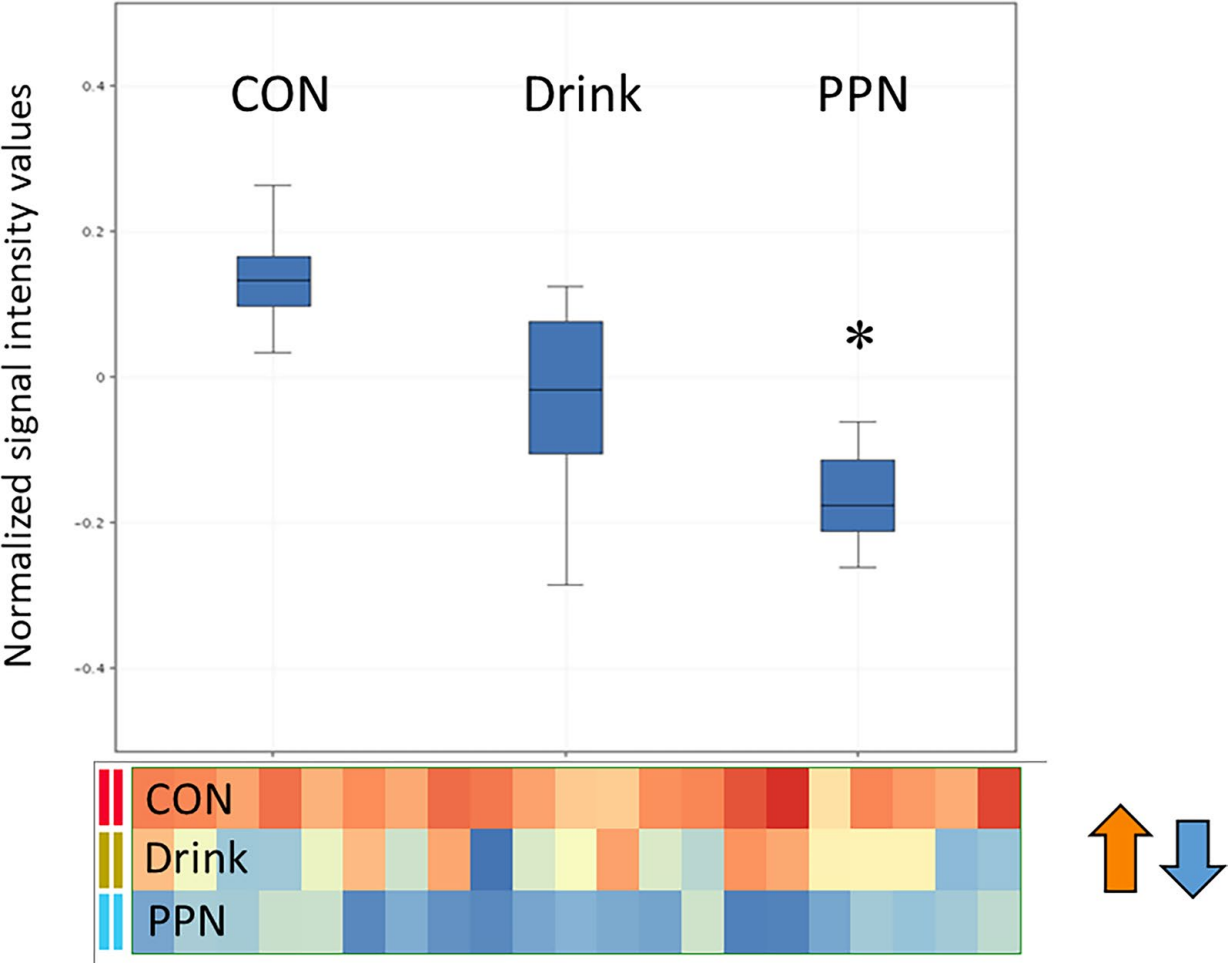
PPN provision down-regulated transcripts related to autophagy and lysosomal protein degradation, without significant effects by Drinks, evaluated by Gene Ontology analyses. Mean signal intensity of transcripts in GO category autophagy-lysosomal degradation (*p < 0.05). Signal intensity of individual transcripts in GO category is shown below the box plot

Gene ontology (GO) analyses by algorithm procedures (AI) were used to search for associations to cell components, biological processes, and molecular functions of regulated transcripts in each patient group (Drink, PPN) compared to Controls. Several GO categories with significant enrichment of transcripts reflected broad general cell functions induced by Drinks and PPN. Drinks mainly affected GO categories related to cell compartment, while few GO categories reflected biological processes and molecular functions, such as oxygen transport and muscle cell contraction. PPN induced enrichment of transcripts in GO categories on molecular functions associated with general categories as protein and enzyme binding, while biological processes were associated with protein ubiquitination, phosphorylation processes and autophagy (GO: 0016567,0006793, 0061919), (Fig. 3). Transcript alterations indicated significant differences between the PPN and Drink groups (post-hoc testing as p < 0.05), related to “catabolic cellular processes” (corrected p-value−0.035, 41 transcripts, data not shown).

### Overall transcript alterations in glucose -and protein metabolism

In addition to GO analyses, transcripts related to glucose metabolism and protein synthesis were manually selected from the list of altered transcripts (ANOVA among groups, p < 0.05, Table 5). Drinks and PPN induced similar directional change compared to Controls in cellular processes as glycolysis (PFKM), glycogen metabolism (GYS1), and glucose transportation (VPS13A and TBC1D1). None of the transcripts related to glucose metabolism were significantly altered between Drink and PPN groups (p > 0.05, Table 5).

**Table 5:** Transcript alterations related to glucose metabolism and protein synthesis

Gene name	Gene symbol	Drink vs. control	PPN vs. control	PPN vs. drink	Function/process	Gene bank accession number
*Glucose metabolism*						
glucose-6-phosphatase catalytic subunit 3	G6PC3	−1.2	−1.0	1.2		NM_138387
glycogen synthase 1	GYS1	**−1.2**	−1.1	1.0	Adds glucose monomers to glycogen	NM_002103
phosphofructokinase, muscle	PFKM	−1.2	**−1.2**	1.0	Catalyzes first committed step in glycolysis	NM_000289
TBC1 domain family member 1	TBC1D1	**−1.8**	−1.4	1.3	GLUT4 translocation	NM_015173
vacuolar protein sorting 13 homolog A	VPS13A	−1.1	**−1.2**	−1.1	GLUT4 translocation	NM_015186
*Protein synthesis*						
eukaryotic translation initiation factor 2 alpha kinase 2	EIF2AK2	**1.13**	−1.06	**−1.19**	Repression of protein synthesis	NM_002759
eukaryotic translation initiation factor 2 alpha kinase 2	EIF2AK2	1.02	**−1.14**	**−1.16**	Repression of protein synthesis	NM_001135652
eukaryotic translation elongation factor 2	EEF2	−1.07	**−1.17**	−1.09	t-rna A-to P-site move	NM_001961
eukaryotic translation elongation factor 1 gamma	EEF1G	**−1.17**	−1.10	1.06	Delivery of t-rna to ribosome	NM_001404
SRP receptor subunit alpha	SRPRA	**−1.17**	1.06	**1.23**	Recognition of signal sequence	NM_003139
l eucyl-tRNA synthetase 2, mitochondrial	LARS2	1.08	**1.24**	1.15	Couple leucine to t-RNA	NM_015340
arginyl-tRNA synthetase 2, mitochondrial	RARS2	−1.03	**−1.16**	**−1.12**	Couple arginine to t-RNA	NM_020320
mitochondrial tRNA translation optimization 1	MTO1	−1.11	1.06	**1.17**	Control of mitochondrial translation	NM_012123
unc-51 like autophagy activating kinase 1	ULK1	−1.12	**−1.43**	**−1.28**	Inhibit mTOR/activate autophagy	NM_003565
unc-51 like autophagy activating kinase 2	ULK2	−1.36	**−1.33**	1.03	Inhibit mTOR/activate autophagy	NM_014683

(ANOVA among groups, p < 0.05). Statistically significant difference between treatment groups (Student-Newman Keuls post hoc p < 0.05) are indicated by bold font. Positive and negative values indicate up-vs down-regulation

By contrast, several transcripts, related to protein synthesis, were significantly altered between PPN and Drink patients (p < 0.05–0.01); as well as compared to control patients (p < 0.05–0.0001, Table 5). Transcript alterations included factors needed in protein translational activities (LARS2, RARS2, SRPRA2, EEF1, EEF2), and signaling molecules for net protein synthesis (EIF2AK2, ULK1). Indeed, PPN reduced the expressions of general repressors of protein synthesis, and activators of autophagy (EIF2AK2; ULK1) compared to Drinks. This is a highly important observation indicating that processes related to both synthesis and degradation of proteins were altered to improve protein anabolism by PPN.

## Discussion

Preoperative carbohydrate loading is worldwide established in recommended treatment protocols for enhanced recovery after surgery (ERAS) [1–3]. The introduction of such guidelines has been constructed to improve quality- and safety of surgical procedures in general, with aims to reduce metabolic stress and support functions of vital organ systems. A main part of various enhanced recovery guidelines is provision of preoperative carbohydrates, both as a nutritional component and a metabolic regulator, although with low evidence [1–3]. Originally this concept emerged to counteract insulin resistance following “preoperative starvation”, and to reduce adrenergic-related stress induction during surgery [22]. This compelling theory has stimulated researchers to evaluate outcomes in both randomized and uncontrolled protocols in thoracic-, abdominal- and orthopedic surgery [4, 5]. Such studies mainly used clinical endpoint variables, with conclusions of minimal effects to high level of evidence by use of carbohydrate drinks before surgery [5]. Some reports even suggested remarkable patient improvements and recovery [14].

A broad spectrum of conclusions may have investigative explanations, but may also be explained by the fact that reported studies were based on insensitive methodology to evaluate detailed metabolic effects in organ systems; where “insulin resistance” may either represent a true whole-body adaptation to decreased assimilation of glucose or altered short-term kinetics of blood insulin and glucose levels only. The aim of the present study was therefore to challenge the question whether a well-recognized Drink nutrition regimen, (ProvideXtra^®^Drink), compares to preoperative peripheral standard total parenteral nutrition (12 h), to induce anabolism in skeletal muscles in cancer patients who experienced weight loss and were aimed at radical surgery. Whole body energy metabolism should then switch from fat oxidation to carbohydrate utilization for ATP production [23, 24]. Our present focus is related to investigate proof-of-concept and not clinical outcomes: that preoperative carbohydrate load may reduce protein breakdown, while muscle protein synthesis is additionally activated by the continuous provision of amino acids during PPN [17, 19, 20], according to our previous translational studies in weight-losing cancer patients, [17–19, 25, 26].

All components and treatment regimens in the present study agreed with routine surgical practice at Sahlgrenska University Hospital, Gothenburg, Sweden, where oral pre-operative nutrition Drinks (ProvideXtra^®^ Drink) are routinely used before elective surgery according to ERAS protocols [1–3]. Our application of peripheral total parenteral nutrition (PPN) has been frequently used as alternative to central venous TPN. Practical aspects on blood and muscle tissue sampling have been applied and confirmed in combinations with modern surgical and anesthesiology procedures [17, 18, 27]. Clinical characteristics show that our patients are well stratified by our computerized algorithm for randomization [16], without any significant differences among the patients groups (Table 1). Preoperative weight losses (5–6%) were comparable among patients, within normal body weight index. None of the patients had clinical or biochemical signs of overt diabetes; and their per-operative insulin levels were within normal levels for healthy age-matched controls (5–19 mIE/L). However, elderly weight-losing cancer patients are usually insulin resistant related to loss of appetite and systemic inflammation [28, 29].

Arterial glucose and serum insulin levels increased significantly by PPN as expected, while increased blood substrate levels either declined rapidly or were insufficiently provided to patients on Drinks; thus a condition without continuous metabolic support to Drink-patients. This is an important difference compared to well recognized findings in our earlier studies on central total parenteral nutrition [21]; with clear cut stimulation of translation initiations of muscle proteins [17]; and altered intracellular signaling [18], as confirmed in the present study. Such anabolic effects depend on both insulin and elevated amino acid concentrations in arterial blood [25, 30]. Normal triglyceride- and urea levels in arterial blood implied that net whole body energy metabolism and substrate oxidation were not changed by either Drinks or PPN [31], although PPN had clear effects on skeletal muscle metabolism.

Importantly, total amino acid concentrations increased 85% by PPN without any change in patients on Drinks compared to Controls; a considerable limitation related to carbohydrate drinks. The Drinks used in the present protocol (ProvideXtra^®^ Drink) contains hydrolyzed proteins corresponding to approximately fifty percent of the amino acid content by continuous PPN infusions (Appendix 1). Provided Drinks did not result in elevated arterial amino acid levels at the start of surgery, in contrast to our previous results that “provision kinetics” of substrates are highly important for net effects on subsequent metabolism [21, 30–32]. Elevated arterial levels of amino acids provide substrates for oxidation and continued protein synthesis; and are possibly local inducers of ectopic anabolic hormone production (estrogens, IGF-1) in skeletal muscles [33, 34]. Sub-optimal conditions for protein anabolism during long-term TPN may be unchanged arterial levels of Tyrosine, due to its low solubility in manufactured intravenous formulas [31], despite our present- and previous results, with improved alterations of intracellular signaling pathways for protein anabolism by overnight standard PPN [17, 18, 27].

In the present study, muscle integrated mRNA transcript analyses (Gene Ontology) showed suppressions of lysosomal and autophagic genes by PPN provision, but not by Drinks as expected (Fig. 3). Also, transcripts directly involved in translation initiation of proteins, showed significant differences between patients on Drink or PPN, compared to Controls (Table 5). Eukaryotic translation initiation factor 2 alpha kinase (EIF2AK2), a global repressor of protein synthesis, was significantly increased in patients on Drinks, but was downregulated by PPN, compared to Controls. Transcript levels of SRP receptor subunit alpha (SRPRA) became upregulated by PPN compared to Drinks. (SRPRA is a subunit of the signal recognition particle, which binds to the signal peptide of nascent protein-chains before attachment to ribosomes on ER membranes). Additional alterations include transcripts related to t-RNA processing and nutrient dependent mTOR signaling (ULK1/ULK2), which induce cells to switch from protein synthesis to activated autophagy [35]. Thus, observed combined alterations are consistent with increased muscle protein synthesis by overnight preoperative PPN; effects that were not observed in muscles from patients on Drinks. However, provision of Drinks or PPN induced similar alterations of glucose metabolism (Table 5). Such, transcript alterations imply increased glucose transport into skeletal muscle (TBC1D1 and VPS13A down). TBC1D1 as well as VPS13A are involved in translocation of glucose transporters from intracellular storage vesicles to become available at the plasma membrane to increase cellular uptake of glucose [36]. Animal experiments imply that at least VPS13A acts to prevent translocation of glucose transporters [36]. Thus, reduced expression of VPS13A may reflect increased availability of glucose transporters at plasma membranes, consistent with previous reports of increased insulin sensitivity following carbohydrate provision [22]. Surprisingly, indications of reduced glycogen synthesis (GYS1 down, Table 5) and reduced glycolysis (PFKM down, Table 5) occurred by both PPN and Drinks. This may suggest elevated glucose flux through the pentose-phosphate shunt, with increased non-oxidative ATP-synthesis, which seems reasonable in resting muscles. Our results imply that provision of glucose, by either preoperative Drinks or PPN, increased muscle glucose metabolism.

Our study design based on transcriptomics may have limitations, since it does not measure dynamics of metabolism such as substrate flux and metabolites. Also, many metabolic events are not directly regulated by gene transcription, particularly in acute physiological conditions. However, complete analyses of the muscle transcriptome (GO) indicate strong evidence of metabolic state and balance among signal pathways for specific metabolic processes at any investigative condition. Thus, a clear-cut difference between PPN and glucose loading in the present study, with shifts towards anabolic alterations of the transcript network, support that Gene Ontology is a possibility to predict or indicate metabolic effects in translational investigations.

In conclusion, published reports have suggested that pre-operative carbohydrate loading is effective to counteract protein catabolism, particularly in muscles; and perhaps to improve muscle energy homeostasis with or without effects on “insulin resistance”. However, our present study implies opposite conclusions: that increased peripheral uptake of glucose by incomplete nutrition (Drinks) may even cause ineffective glucose utilization by non-oxidative pathways without improvement of net muscle protein balance. Overnight continuous PPN seems to stimulate muscle protein synthesis according to GO-analyses, as shown in our previous studies [17–19, 25, 26], in combination with decreased lysosomal degradation. Thus, our present study shows that pre-operative PPN is an outstanding alternative to support nutritionally deprived cancer patients aimed at surgical procedures, while oral glucose loading did not indicate muscle anabolism and should therefore not be recommended in combination with major cancer surgery.

## Data Availability

All data generated or analyzed during this study are included in this published article. The datasets that support the findings of this study are not openly available due to reasons of sensitivity and are available from the corresponding author upon reasonable request.

## Appendix 1

Supplemental information to be published electronically

See Table 6.

**Table 6:** Compositions of ProvideXtra Drink^®^ and SmofKabiven perifer^®^ according to specifications by the manufacturer (Fresenius Kabi, Sweden)

Product content	ProvideXtra drink per 600 ml total amount in evening and morning dose	Smof Kabiven Perifer amount per 12 h infusion and 70 kg body weight
Carbohydrates	201 g (maltodextrin 148 g, sucrose 53 g)	99 g (glucose)
Fat	0 g	39,5 g
Hydrolyzed Proteins or amino acids	24 g^a^	44.2 g^b^
Calculated Energy content, kcal:		
Total energy	900 kcal^c^	980 kcal^d^
Total non-protein kcal (carbohydrates, fat)	804 kcal	840 kcal

^a^0.35 g/kg

^b^0.63 g/kg

^c^13 kcal/kg

^d^14 kcal/kg

all numbers (a–d) with a variation of ± (3–4) %

## References

[1] Gustafsson UO, Scott MJ, Hubner M, Nygren J, Demartines N, Francis N, et al. Guidelines for perioperative care in elective colorectal surgery: enhanced recovery after surgery (ERAS((R))) society recommendations: 2018. World J Surg. 2019;43(3):659–95. 30426190 10.1007/s00268-018-4844-y

[2] Mortensen K, Nilsson M, Slim K, Schafer M, Mariette C, Braga M, et al. consensus guidelines for enhanced recovery after gastrectomy: enhanced recovery after surgery (ERAS(R)) society recommendations. Br J Surg. 2014;101(10):1209–29. 25047143 10.1002/bjs.9582

[3] Melloul E, Lassen K, Roulin D, Grass F, Perinel J, Adham M, et al. Guidelines for perioperative care for pancreatoduodenectomy: enhanced recovery after surgery (ERAS) recommendations 2019. World J Surg. 2020;44(7):2056–84. 32161987 10.1007/s00268-020-05462-w

[4] Amer MA, Smith MD, Herbison GP, Plank LD, McCall JL. Network meta-analysis of the effect of preoperative carbohydrate loading on recovery after elective surgery. Br J Surg. 2017;104(3):187–97. 28000931 10.1002/bjs.10408

[5] Ricci C, Ingaldi C, Alberici L, Serbassi F, Pagano N, De Raffele E, et al. Preoperative carbohydrate loading before elective abdominal surgery: a systematic review and network meta-analysis of phase II/III randomized controlled trials. Clin Nutr. 2022;41(2):313–20. 34999325 10.1016/j.clnu.2021.12.016

[6] Ricci C, Ingaldi C, Alberici L, Serbassi F, Pagano N, De Raffele E, et al. Preoperative carbohydrate loading before elective abdominal surgery: a systematic review and network meta-analysis of phase II/III randomized controlled trials. Clin Nutr. 2021;41(2):313–20. 34999325 10.1016/j.clnu.2021.12.016

[7] Pillinger NL, Robson JL, Kam P. Nutritional prehabilitation: physiological basis and clinical evidence. Anaesth Intensive Care. 2018;46(5):453–62. 30189818 10.1177/0310057X1804600505

[8] Nygren J, Soop M, Thorell A, Efendic S, Nair KS, Ljungqvist O. Preoperative oral carbohydrate administration reduces postoperative insulin resistance. Clin Nutr. 1998;17(2):65–71. 10205319 10.1016/S0261-5614(98)80307-5

[9] Nygren J, Soop M, Thorell A, Sree Nair K, Ljungqvist O. Preoperative oral carbohydrates and postoperative insulin resistance. Clin Nutr. 1999;18(2):117–20. 10459075 10.1016/S0261-5614(99)80063-6

[10] Shi M, Hu Z, Yang D, Cai Q, Zhu Z. Preoperative oral carbohydrate reduces postoperative insulin resistance by activating AMP-activated protein kinase after colorectal surgery. Dig Surg. 2020;37(5):368–75. 32155622 10.1159/000505515

[11] Tewari N, Awad S, Duska F, Williams JP, Bennett A, Macdonald IA, et al. Postoperative inflammation and insulin resistance in relation to body composition, adiposity and carbohydrate treatment: a randomised controlled study. Clin Nutr. 2019;38(1):204–12. 29454501 10.1016/j.clnu.2018.01.032 PMC6380471

[12] Svanfeldt M, Thorell A, Hausel J, Soop M, Rooyackers O, Nygren J, et al. Randomized clinical trial of the effect of preoperative oral carbohydrate treatment on postoperative whole-body protein and glucose kinetics. Br J Surg. 2007;94(11):1342–50. 17902094 10.1002/bjs.5919

[13] Crowe PJ, Dennison A, Royle GT. The effect of pre-operative glucose loading on postoperative nitrogen metabolism. Br J Surg. 1984;71(8):635–7. 6430379 10.1002/bjs.1800710828

[14] Henriksen MG, Hessov I, Dela F, Hansen HV, Haraldsted V, Rodt SA. Effects of preoperative oral carbohydrates and peptides on postoperative endocrine response, mobilization, nutrition and muscle function in abdominal surgery. Acta Anaesthesiol Scand. 2003;47(2):191–9. 12631049 10.1034/j.1399-6576.2003.00047.x

[15] Dock-Nascimento DB, de Aguilar-Nascimento JE, Magalhaes Faria MS, Caporossi C, Slhessarenko N, Waitzberg DL. Evaluation of the effects of a preoperative 2-hour fast with maltodextrine and glutamine on insulin resistance, acute-phase response, nitrogen balance, and serum glutathione after laparoscopic cholecystectomy: a controlled randomized trial. JPEN J Parenter Enteral Nutr. 2012;36(1):43–52. 22235107 10.1177/0148607111422719

[16] Pocock SJ, Simon R. Sequential treatment assignment with balancing for prognostic factors in the controlled clinical trial. Biometrics. 1975;31(1):103–15. 1100130 10.2307/2529712

[17] Iresjo BM, Korner U, Hyltander A, Ljungman D, Lundholm K. Initiation factors for translation of proteins in the rectus abdominis muscle from patients on overnight standard parenteral nutrition before surgery. Clin Sci. 2008;114(9–10):603–10. 10.1042/CS20070359 18001269

[18] Iresjo BM, Engstrom C, Lundholm K. Preoperative overnight parenteral nutrition (TPN) improves skeletal muscle protein metabolism indicated by microarray algorithm analyses in a randomized trial. Physiol Rep. 2016. 10.14814/phy2.12789. 27273879 10.14814/phy2.12789 PMC4908486

[19] Lundholm K, Schersten T. Protein synthesis in human skeletal muscle tissue: influence of insulin and amino acids. Eur J Clin Invest. 1977;7(6):531–6. 415875 10.1111/j.1365-2362.1977.tb01647.x

[20] Iresjo BM, Svanberg E, Lundholm K. Reevaluation of amino acid stimulation of protein synthesis in murine- and human-derived skeletal muscle cells assessed by independent techniques. Am J Physiol Endocrinol Metab. 2005;288(5):E1028–37. 15598673 10.1152/ajpendo.00295.2004

[21] Hyltander A, Warnold I, Eden E, Lundholm K. Effect on whole-body protein synthesis after institution of intravenous nutrition in cancer and non-cancer patients who lose weight. Eur J Cancer. 1991;27(1):16–21. 1826434 10.1016/0277-5379(91)90051-E

[22] Ljungqvist O. Modulating postoperative insulin resistance by preoperative carbohydrate loading. Best Pract Res Clin Anaesthesiol. 2009;23(4):401–9. 20108579 10.1016/j.bpa.2009.08.004

[23] Awad S, Constantin-Teodosiu D, Constantin D, Rowlands BJ, Fearon KC, Macdonald IA, et al. Cellular mechanisms underlying the protective effects of preoperative feeding: a randomized study investigating muscle and liver glycogen content, mitochondrial function, gene and protein expression. Ann Surg. 2010;252(2):247–53. 20622656 10.1097/SLA.0b013e3181e8fbe6

[24] Webber J, Taylor J, Greathead H, Dawson J, Buttery PJ, Macdonald IA. Effects of fasting on fatty acid kinetics and on the cardiovascular, thermogenic and metabolic responses to the glucose clamp. Clin Sci (Lond). 1994;87(6):697–706. 7874862 10.1042/cs0870697

[25] Moller-Loswick AC, Zachrisson H, Hyltander A, Korner U, Matthews DE, Lundholm K. Insulin selectively attenuates breakdown of nonmyofibrillar proteins in peripheral tissues of normal men. Am J Physiol. 1994;266(4 Pt 1):E645–52. 8178986 10.1152/ajpendo.1994.266.4.E645

[26] Mollerloswick AC, Zachrisson H, Bennegard K, Sandstrom R, Lundholm K. Insufficient effect of total parenteral-nutrition to improve protein balance in peripheral-tissues of surgical patients. Jpen-Parenter Enter. 1991;15(6):669–75. 10.1177/0148607191015006669 1766058

[27] Iresjo BM, Engstrom C, Smedh U, Lundholm K. Overnight steady-state infusions of parenteral nutrition on myosin heavy chain transcripts in rectus abdominis muscle related to amino acid transporters, insulin-like growth factor 1, and blood amino acids in patients aimed at major surgery. JPEN J Parenter Enteral Nutr. 2019;43(4):497–507. 30350380 10.1002/jpen.1458

[28] Eden E, Edstrom S, Bennegard K, Schersten T, Lundholm K. Glucose flux in relation to energy expenditure in malnourished patients with and without cancer during periods of fasting and feeding. Cancer Res. 1984;44(4):1718–24. 6367972

[29] Lundholm K, Holm G, Schersten T. Insulin resistance in patients with cancer. Cancer Res. 1978;38(12):4665–70. 719645

[30] Svanberg E, Moller-Loswick AC, Matthews DE, Korner U, Andersson M, Lundholm K. Effects of amino acids on synthesis and degradation of skeletal muscle proteins in humans. Am J Physiol. 1996;271(4 Pt 1):E718–24. 8897860 10.1152/ajpendo.1996.271.4.E718

[31] Iresjo BM, Korner U, Larsson B, Henriksson BA, Lundholm K. Appearance of individual amino acid concentrations in arterial blood during steady-state infusions of different amino acid formulations to ICU patients in support of whole-body protein metabolism. JPEN J Parenter Enteral Nutr. 2006;30(4):277–85. 16804124 10.1177/0148607106030004277

[32] Lundholm K, Bennegard K, Zachrisson H, Lundgren F, Eden E, Moller-Loswick AC. Transport kinetics of amino acids across the resting human leg. J Clin Invest. 1987;80(3):763–71. 3624488 10.1172/JCI113132 PMC442301

[33] Iresjo BM, Landin A, Ohlsson C, Lundholm K. Estrogen biosynthesis in cultured skeletal muscle cells (L6) induced by amino acids. Genes Nutr. 2019;14:29. 31741685 10.1186/s12263-019-0652-8 PMC6849273

[34] Iresjo BM, Diep L, Lundholm K. Initiation of muscle protein synthesis was unrelated to simultaneously upregulated local production of IGF-1 by amino acids in non-proliferating L6 muscle cells. PLoS ONE. 2022;17(7): e0270927. 35802556 10.1371/journal.pone.0270927 PMC9269383

[35] Nazio F, Cecconi F. Autophagy up and down by outsmarting the incredible ULK. Autophagy. 2017;13(5):967–8. 28282264 10.1080/15548627.2017.1285473 PMC5446071

[36] Hook SC, Chadt A, Heesom KJ, Kishida S, Al-Hasani H, Tavare JM, et al. TBC1D1 interacting proteins, VPS13A and VPS13C, regulate GLUT4 homeostasis in C2C12 myotubes. Sci Rep. 2020;10(1):17953. 33087848 10.1038/s41598-020-74661-1 PMC7578007

